# Feeding Behavior of Fattening Bulls Fed Six Times per Day Using an Automatic Feeding System

**DOI:** 10.3389/fvets.2020.00043

**Published:** 2020-02-05

**Authors:** Laura Schneider, Nina Volkmann, Nicole Kemper, Birgit Spindler

**Affiliations:** Institute for Animal Hygiene, Animal Welfare and Animal Behavior, University of Veterinary Medicine Hannover, Foundation, Hanover, Germany

**Keywords:** automatic feeding, animal behavior, fattening cattle, eating behavior, feeding frequency

## Abstract

The usage of automatic feeding systems (AFS) in cattle offers multiple advantages, mostly due to the possibility of an increased feeding frequency. While it is gaining more and more importance in dairy farming, there is still a lack of experience and scientific knowledge regarding its use in fattening cattle. The aim of this study was to describe the behavior of 56 Simmental bulls fed with an AFS six times daily a total mixed ration. The animals arrived at the farm with an average age of 148 ± 11 days. They were housed in four straw-bedded pens in groups of 14 animals each. Their average slaughter age was 558 ± 20 days. Behavioral observations were made during three observation periods (OP) at an average of 11, 14, and 16 months of age. Using scan sampling, feeding, and lying behavior of all animals and the order of bulls feeding after feed delivery were recorded. Furthermore, body condition and health status were monitored and complemented with the carcass weights. Body condition, health status, and carcass weights of the bulls were found to be satisfactory: Mean body condition score increased from 2.8 ± 0.3 in OP1 to 3.0 ± 0.1 in OP3 and mean carcass weight was 432.71 ± 40.82 kg. No severe health problems occurred. The feeding activity of the bulls was spread out over the course of the day with peaks in the afternoon and evening. Percentages of bulls feeding per pen never exceeded 20%, animals feeding mostly alone (during 28.04 ± 2.15% of total observation time) or in groups of two to three (16.61 ± 2.00% and 6.74 ± 1.90%). The order of bulls feeding after feed delivery varied indicating that all animals had similar access to fresh feed. These results emphasize the importance of constant feed availability and quality at any time of the day, thus indicating the ability of an AFS with six daily feedings to ensure such a consistency.

## Introduction

Automatic feeding is gaining more and more importance in dairy cattle farming. Main reasons to install an automatic feeding system (AFS) are its contributions to a reduction in workload and working time as well as increasing flexibility (1). However, benefits of AFS are not restricted to management factors. Permitting an increasing feeding frequency without increasing costs or workload they also offer possibilities to positively influence behavior (2, 3), productivity (4, 5), and health of cattle (2, 4, 5).

Up to now, AFS have been rarely used in housing systems for fattening cattle, despite their advantages being transferable from dairy to fattening cattle. Fattening cattle are commonly fed twice per day, in the morning and in the evening or even only once in the morning, using an *ad libitum* feeding regimen. On pasture, cattle spend about 10–12 h per day grazing, divided into several meals spread out from dusk to dawn (6). The feeding duration of housed cattle is reduced to 4–7 h per day, but feeding is still divided into 6–12 daily meals spread out over the daylight period (6, 7). As cattle are selective feeders while consuming conserved feed (6), feed composition, palatability and, therefore, possibly also feed quality is likely to decline with increasing time after feed delivery. This could have detrimental effects on nutrition, as cattle tend to spread out their feeding behavior over the course of the day. The pre-selected ration they receive with increasing time after feeding may not fulfill their nutrient requirements. An increasing frequency of feed deliveries spread out across the day could contribute to ensuring not only constant feed availability but also constant feed quality. Using an AFS allows such a feed delivery pattern without increasing workload for farmers.

Further positive effects of an increased feeding frequency are known from studies on dairy cattle regarding an increase in the time spent feeding (2, 3, 8) as well as in dry matter intake (4, 9). Furthermore, increasing feeding frequency led to decreased diurnal variation in ruminal pH possibly reducing the risk of subacute ruminal acidosis (4, 5, 10). However, all these results refer to dairy cattle and heifers, while currently no scientific studies at all exist concerning the influence of feeding frequency on behavior, health, or growth performance of fattening cattle. Nonetheless, the impact of feeding frequency is not only important regarding the suitability or benefits of an AFS for fattening cattle, but also concerning the current practice of feeding fattening cattle only once or twice per day. Therefore, the objective of the present study was to generally describe behavior and performance of fattening bulls fed at a high frequency, namely six times per day using an AFS.

## Materials and Methods

### Animals, Housing and Management

This study did not involve a prospective evaluation or laboratory animals and only non-invasive procedures were applied. It was reviewed and it received approval from the Animal Welfare Officer of the University of Veterinary Medicine Hannover, Foundation (reference: TVO-2017-B5). The study was carried out in accordance with the German legislations, the German Animal Welfare Act [German designation: TierSchG (11)], national requirements for animal husbandry [German designation: TierSchNutztV (12)], the Animal Protection guideline for Fattening Cattle of Lower Saxony (13) as well as the Council of Europe Convention on the protection of animals kept for farming purposes and its recommendations concerning cattle (14). The study was conducted on 56 Simmental bulls on a commercial fattening farm in Lower Saxony, Germany, housing 336 fattening bulls in total. The bulls were housed in four groups of 14 animals each in straw-bedded pens with a space allowance of 4 m^2^ per bull. They were automatically fed six times per day a total mixed ration with an AFS (feeding robot Triomatic HP 2 300, Trioliet, Oldenzaal, the Netherlands). The number of six feed deliveries was chosen according to Oberschätzl-Kopp et al. (3). Feed delivery occurred around 6:15, 10:15, 14:15, 18:15, 19:15, and 22:15 h. In addition, feed was pushed-up toward the feed barrier by the feed mixing and distribution wagon two times daily (around 13:15 and 21:15 h) and residues were removed manually once per day at 17:30 h. The feeding area was not provided with feeding gates or head barriers and the manger space was 4.85 m per pen. The feed composition and particle size distribution of the diet is reported in Table 1. Water was available *ad libitum* via two drinking troughs per pen located on both sides in the middle of the pen. Approximately 3 kg straw per animal were distributed daily around 17:00 h with a straw blower.

**Table 1:** Ingredients, chemical composition [related to dry matter (DM)] and particle size distribution of the total mixed ration.

**INGREDIENT [%]**	
Maize silage	72.53
Potato pulp	12.73
Rye with calcium carbonate and salt	5.82
Rapeseed meal	3.88
Soybean meal	3.48
Barley straw	0.97
Mineral and vitamin mix	0.58
**CHEMICAL COMPOSITION**	
Dry matter [%]	40.5
Crude protein [% DM]	12.9
Crude ash [% DM]	6.1
Crude fat [% DM]	2.3
Crude fiber [% DM]	16.1
Nitrogen free extractives [% DM]	62.6
pH	4.62
**PARTICLE SIZE DISTRIBUTION [%]**	
Particles retained by the 19 mm sieve (long)	10.93
Particles retained by the 8 mm sieve (medium)	50.49
Particles on the bottom pen (short)	38.58

The animals arrived on the farm at about 5 months of age (148 ± 11 days). In the following 3 months, they were assigned to groups of 14 animals each according to their body weight. For the study, four groups of similar body weight were selected. These groups remained constant until the end of the fattening period. The only exception was group 3 from which one animal (animal 32) was removed after the first observation period (OP), explained below, due to reduced feeding and growth. Animals were slaughtered at an age of 558 ± 20 days and carcass weights were provided by the slaughterhouse.

### Body Condition and Health Scoring

The data acquisition began when the final groups had existed for at least 3 weeks. It was performed during three OP at an average of 338, 407, and 476 days of age, respectively (approximately at an age of 11, 14, and 16 months, respectively). The OP was defined as a 4 day-period of data acquisition, beginning with body condition and health scoring on the first day and continuing with 3 days of continuous video recording at the following 3 days. At the beginning of each OP, all individual animals were individually scored for body condition and health status by one trained observer. The bulls' body condition was assessed following the body condition score (BCS) system described by Edmonson et al. (15) with scores ranging from 1 (emaciated condition) to 5 (obese condition) in steps of 0.5 (Table 2). Bulls' health status was assessed considering the welfare criteria for health assessment listed in the Welfare Quality® assessment protocol for cattle (16). The analyzed criteria are listed in Table 3. In addition to observations during scoring, bulls' health was monitored by the farmer. In consultation with his farm veterinarian, he documented any pathological event that occurred during the fattening period as well as drug use.

**Table 2:** Body condition scoring system described by Edmonson et al. (15).

**Score**	**Body condition**
1	Severe underconditioning (emaciated)
2	Frame obvious
3	Frame and covering well-balanced
4	Frame not as visible as covering
5	Severe overconditioning (obese)

**Table 3:** Welfare criteria concerning health following the Welfare Quality® assessment protocol for cattle (16).

**Welfare criteria**	**Measures**	**Description**
Absence of injuries	Lameness	Abnormality of movement
	Integument alterations	Hairless patches and lesions/swellings
Absence of disease	Coughing	Sudden and noisy expulsion of air from the lungs
	Nasal/ocular discharge	Clearly visible flow/discharge from nostrils/eye
	Hampered respiration	Deep and overtly difficult or labored breathing
	Bloated rumen	“Bulge” between hip bone and ribs on the left side
	Diarrhea	Loose watery manure below tail head on both sides of the tail

### Behavioral Observations

Behavioral observations were performed by analyzing video recordings. The animals were videotaped with two video cameras (EQ900F, EverFocus Electronics Corporation, Taipei, Taiwan) and an eight-channel hybrid recorder (AXR-108, Monacor International GmbH & Co. KG, Bremen, Germany). The cameras were located above the pens covering two full pens each. Individual animals were identified and listed by the color and patterns of their fur.

The video analyses were performed using the program Interact (Version 17.0.1.2, Mangold International GmbH, Arnstorf, Germany) for observational research. For the group-based evaluation, the activity of the animals was observed for 48 h per OP using a scan-sampling-technique (17). In intervals of 2 min from 4:00 to 23:30 h and 10 min during the night (23:30–4:00 h), the number of animals feeding and lying was recorded. At an individual level, the behavior of all animals was scanned at intervals of 10 min from 4:00 to 22:30 h on 3 consecutive days per OP using a combination of scan sampling and focal animal sampling (17). The 10 min-interval was chosen in accordance with Mitlohner et al. (18) and Endres et al. (19). The recorded and analyzed behavior patterns were feeding and lying. Lying included bulls that were observed in sternal as well as in total lateral recumbency from the end of the lying-down movement until the end of the standing-up movement. An animal was considered to be feeding when its head was completely past the feed rail and above the feed while ingesting the feed.

### Behavioral Analysis

In accordance with Endres et al. (19), each behavior was assumed to persist for the entire sample interval. Therefore, the duration of each performed behavioral pattern was calculated by multiplying the number of the correspondent sample intervals with 2 or 10. For the behavioral observations at herd level, the percentage of animals performing each behavior was averaged for each interval for all days. To assess behavioral synchronization the percentage of time a certain number of animals spent feeding or lying was averaged for all groups and days. The variables calculated per animal at individual level are listed in Table 4. Mean and SD of the percentage of time spent feeding and lying per 18.5 h-period were calculated for each single animal for all days. Furthermore, the mean bout length and the mean number of bouts per 18.5 h-period were determined. After each observed feed delivery, the position of each animal within the order of animals feeding after feed delivery was determined. Furthermore, for each single animal, the percentage of feed deliveries with following feeding behavior was calculated.

**Table 4:** Behavioral observations at individual level: Variables calculated per animal.

**Behavior**	**Variable**	
Lying	Mean percentage of time spent lying	Per 18.5 h-period^a^
	Mean number of lying bouts	
	Mean lying bout length	
Feeding	Mean percentage of time spent feeding	Per 18.5 h-period^a^
	Mean number of feeding bouts	
	Mean feeding bout length	
	Mean feeding order position^b^	Averaged for 54 observed feed deliveries^d^
	Percentage of feed deliveries with following feeding activity^c^	

a *Averaged for three observation periods with three 18.5 h-periods of observation each*.

b *After each observed feed delivery, the position of each animal within the order of animals feeding after feed delivery was determined. Bulls feeding for the first time after feed delivery at the same interval received the same feeding order position value*.

c *Feed delivery with following feeding activity means that an animal was observed feeding before the beginning of the next feed delivery or the end of the 18.5 h-period*.

d *Three observation periods of 3 days each and six feed deliveries per day*.

### Statistical Analysis

Statistical analyses were performed using SAS 9.4 (SAS Institute Inc., Cary, NC, USA, 1999). First, a descriptive analysis was performed to show frequency distributions and averages. Subsequently, the dependent variables of percentage of time spent lying and feeding as well as mean bout length and mean number of lying and feeding bouts per 18.5 h-period were tested for normal distribution using histograms and a Shapiro-Wilk test to determine a suitable statistical model for the evaluation. As data were non-normally distributed, a one-way ANOVA on ranks (Kruskal-Wallis Test) was conducted to examine the differences between the individual animals concerning the dependent variables. Correlations between carcass weight and BCS and the dependent variables were tested using Spearman's correlation.

## Results

### Health, Body Condition and Growth Performance

Observed health problems during scoring were incidents of temporary lameness in three bulls and hairless patches (skin not damaged, no lesions or swellings) on neck or forehead in five bulls. Furthermore, one bull (animal 32 in group 3) was removed from the group after the end of OP1 due to reduced growth. Besides, no health problems were observed and no specific medical treatments were required throughout the finishing period. BCS ranged from 2 to 3.5, with mean values increasing from OP1 to OP3 (Table 5). The mean carcass weight of the bulls was 432.7 ± 40.8 kg (Table 5).

**Table 5:** Body Condition Score (BCS) at the different Observation Periods (OP) and carcass weight.

	***n***	**Mean ± SD**	**Minimum**	**Maximum**
BCS OP1	56	2.8 ± 0.3	2	3
BCS OP2	55	2.9 ± 0.2	2.5	3.5
BCS OP3	55	3.0 ± 0.1	2.5	3.5
Carcass weight [kg]	55	432.71 ± 40.82	213.00	489.00

*BCS scoring system described by Edmonson et al. (15)*.

### Behavioral Observations at Herd Level

**Figure 1:**
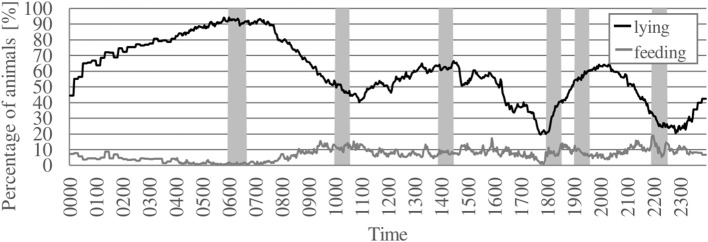
Averaged percentage of animals feeding and lying per pen over a 24 h-period. Black line = percentage of animals lying, gray line = percentage of animals feeding, gray areas = time periods of feed delivery. Data were averaged for each interval for three observation periods with 2 days each and 56 animals housed in four groups of 13–14 animals each.

The averaged percentages of animals feeding and lying during the course of the day are presented in Figure 1. The feeding activity was widely spread out over the course of the day with averaged percentages of animals feeding per pen never exceeding 20%. Maximum values of animals feeding occurred in the afternoon and evening around 16:00 and 22:00 h. Low percentages of animals feeding as well as maximum percentages of more than 90% animals lying occurred during the night and early morning hours (maximum around 06:00 h). Two further lying periods with lower maximum percentages of 60–70% bulls lying were observed in the afternoon and evening (around 14:00 and 20:00 h).

The most frequent feeding condition showed one bull feeding alone, followed by two to three bulls feeding at the same time (Table 6). Simultaneous feeding of more than three bulls was rarely observed. During 45.6 ± 4.0% of total observation time no animals were observed feeding. The most frequent lying conditions were 61–80% of the animals per pen lying simultaneously, closely followed by 41–60% of the animals lying at the same time (Table 7). More than 80% animals lying per pen were observed during 20.48 ± 2.25% of total observation time. Simultaneous lying of all animals per pen (100%) was observed in all pens.

**Table 6:** Number of simultaneous feeding animals and percentage of time.

**Number of bulls feeding simultaneously**	**Percentage of total observation time [%]**		
	**Mean ± SD**	**Minimum**	**Maximum**
0	45.59 ± 4.04	39.31	52.43
1	28.04 ± 2.15	23.89	31.94
2	16.61 ± 2.00	14.03	20.00
3	6.74 ± 1.90	3.75	10.07
4	2.31 ± 1.03	1.32	4.65
5	0.63 ± 0 42	0.14	1.60
6	0.06 ± 0.12	0.00	0.42
7	0.02 ± 0.03	0.00	0.07

*Data were averaged for three observation periods with 2 days (48 h) each and four groups of 13–14 animals each*.

**Table 7:** Percentage of simultaneous lying animals and percentage of time.

**Percentage of bulls lying simultaneously [%]**	**Percentage of total observation time [%]**		
	**Mean ± SD**	**Minimum**	**Maximum**
0	2.68 ± 2.34	0.07	7.36
1–20	4.72 ± 1.82	1.67	7.99
21–40	11.55 ± 4.40	6.67	19.79
41–60	28.94 ± 5.57	19.24	37.43
61–80	31.63 ± 7.15	18.61	38.40
81–100	20.48 ± 2.25	16.32	23.89

*Data were averaged for three observation periods with 2 days (48 h) each and four groups of 13–14 animals each*.

### Behavioral Observations at Individual Level

Each bull spent on average 9.9 ± 1.8% of the 18.5 h-period of individual observation feeding, and 62.7 ± 4.3% lying (Table 8). Per 18.5 h-period each animal had on average 7.9 ± 1.2 feeding bouts with a mean duration of 13.5 ± 1.4 min and 11.0 ± 1.4 lying bouts with a mean duration of 62.9 ± 9.0 min.

**Table 8:** Individual observation results.

**Measurement**	**Mean ± SD**	**Minimum**	**Maximum**
Percentage of time spent feeding per 18.5 h-period [%]	9.9 ± 1.8	6.7	14.4
Percentage of time spent lying per 18.5 h-period [%]	62.7 ± 4.3	51.6	72.6
Percentage of feed deliveries with following feeding [%]	75.9 ± 6.8	61.1	92.6
Mean number of feeding bouts per 18.5 h-period	7.9 ± 1.2	5.2	11.4
Mean number of lying bouts per 18.5 h-period	11.0 ± 1.4	8.1	14.9
Mean feeding bout length [min]	13.5 ± 1.5	11.1	17.4
Mean lying bout length [min]	62.9 ± 9.0	37.4	85.1

*Data were averaged for three observation periods with three 18.5 h-periods of observation each and 56 animals housed in four groups*.

The individual animals showed significant differences concerning their mean percentage of time spent feeding (*p* = 0.0021) and lying (*p* < 0.0001). Furthermore, they differed in their mean number of feeding and lying bouts (feeding: *p* = 0.0039, lying: *p* < 0.0001) as well as the mean bout length (feeding: *p* = 0.0079, lying: *p* = 0.0002). None of these variables were correlated with carcass weight or BCS (Table 9).

**Table 9:** Spearman's correlation of the variables describing feeding and lying behavior with carcass weight and body condition score.

**Variable**	**Carcass weight**		**BCS**	
	**r_**sp**_**	**P**	**r_**sp**_**	**P**
Percentage of time spent feeding per 18.5 h-period	−0.07352	0.5937	−0.20653	0.1267
Percentage of time spent lying per 18.5 h-period	0.14459	0.2922	0.24873	0.0645
Mean number of feeding bouts per 18.5 h-period	−0.02716	0.8440	−0.06923	0.6122
Mean number of lying bouts per 18.5 h-period	0.04524	0.7430	0.02642	0.8467
Mean feeding bout length	0.01342	0.9225	−0.19227	0.1557
Mean lying bout length	0.08079	0.5576	0.08092	0.5533

*BCS, body condition score; r_sp_, Spearman's rank correlation coefficient*.

**Figure 2:**
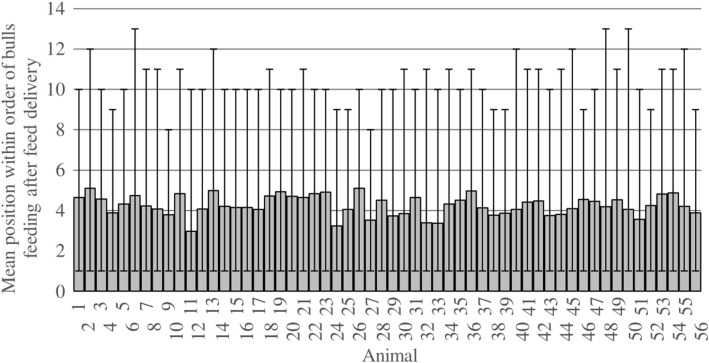
Mean, minimum and maximum positions of the individual bulls within the order of bulls feeding after feed delivery per pen. Data were recorded per pen and averaged per individual for all observed feed deliveries over three observation periods with 3 days each and six feed deliveries per day (*n* = 54); mean position values are marked by bars, minimum and maximum values are marked by whiskers. Animals 1–14 = group 1; animals 15–28 = group 2; animals 29–42 = group 3; animals 43–56 = group 4.

During the observed feed deliveries (*n* = 54), each bull was observed both feeding as the first one after feed delivery and as one of the latter (feeding order position 8–13; Figure 2). With a mean feeding order position of an average of 4.3 ± 0.5, the feeding order varied between feed deliveries. The averaged percentage of feed deliveries with following feeding activity was 75.9 ± 6.8% (Table 8).

## Discussion

Under natural conditions, cattle display a need to spread out their feeding behavior over the whole day. As diurnal feeders, they perform grazing activity during several meals from dawn to dusk with concentrations at crepuscular times (6, 20). Housed cattle also prefer feeding during the daylight hours. In consistence, the bulls in the present study spread out their feeding behavior over the course of the day with reduced feeding activity during the night and early hours of the morning. Similar results were described by other authors for fattening bulls (21) as well as for lactating dairy cows (22). The feeding behavior of the bulls in the present study was also evenly distributed over the course of the daylight hours, resembling the behavior of cattle under natural conditions: It was divided into an average of 7.9 bouts, each bull feeding on average after more than three quarters of the daily feed deliveries. Furthermore, the maximum number of feeding bulls at any one time was observed during the afternoon and evening. Cozzi and Gottardo (21) made similar observations in housed fattening bulls with peaks in feeding activity occurring after feed delivery, but also around sunset. This resembles another aspect of natural cattle behavior, described by Philipps (6): Cattle on pasture take the longest and most intensive mealtime of the day in the evening, this ending shortly after dusk, providing them with sufficient food to digest during the night. This may also be the case in the present study: The feeding activity in the morning during and after the first feed delivery of the day was quite low, indicating that the bulls accumulated sufficient quantities of feed to digest during the night and, consequently, were not starving after the night hours without a delivery of fresh feed.

It is generally regarded as an indicator of a high level of animal welfare when animals in housing systems display behavior that resembles their natural behavior (23). Furthermore, the displayed feeding behavior may imply beneficial effects on the bull's health: In dairy cattle, several authors describe a decrease in diurnal variation in ruminal pH after increasing the feeding frequency (4, 10). As an explanation, they offer the more even distribution of the feeding activity over the course of the day. The lowered variation in ruminal pH leads to a reduced risk of developing subacute ruminal acidosis (SARA). This was confirmed by the findings of Macmillan et al. (5), with cows at higher risk of SARA feeding at irregular intervals throughout the day. Consistently, in the present study with a high feeding frequency of six daily feed deliveries, no considerable number of digestive disorders occurred. One bull was removed from its group due to reduced growth, but none of the other animals displayed possible signs of digestive disorders like acidosis, which are common problems in fattening cattle (24–27).

The most common health problem in the housing of fattening cattle are respiratory diseases (27–30). Further issues are lameness and tail tip lesions (29–32). However, lameness and tail tip lesions more commonly occur in slatted floor systems with a reduced risk of occurrence in straw-bedded pens (31, 33, 34). In the present study, no diseases occurred and the only observed injuries were temporary lameness and hairless patches on the neck and forehead affecting three and five bulls, respectively, during the entire fattening period. Therefore, the number of injuries was rather low and none of them were severe. The hairless patches were not permanent and not combined with either lesions or swellings and the incidents of lameness were only temporary and disappeared without medical treatment. The overall health status of the bulls was satisfactory. Consistently, with BCS ranging from 2 to 3.5, there were no bulls in an emaciated or obese condition at any time. Mean values increased from 2.8 ± 0.3 in OP1 to 3.0 ± 0.1 in OP3, so over the course of the fattening period the BCS came close to a value of 3, which is consistent with a well-balanced frame and covering (15). Mean carcass weights were consistent with the average carcass weight of Simmental bulls in Lower Saxony (35). Bulls with a lower BCS at the beginning of the fattening period could improve their body condition within the following months, indicating that they did not suffer from restricted access to feed due to their weaker body condition. This was confirmed by the varying order of bulls feeding after feed delivery. Obviously, there were no bulls always feeding first or always feeding last. Consequently, all animals had similar access to fresh and non-preselected feed. The bulls differed significantly from each other concerning their feeding and lying behavior, but none of the analyzed variables were correlated with carcass weight or BCS. Thus, there were no restrictions in feeding or lying behavior leading to restricted growth performance.

As gregarious animals, cattle generally display a highly synchronized feeding behavior (6, 36). However, housed cattle exhibit less social facilitation when feeding than grazing cattle on pasture (6). This could be partly caused by an alternation of synchronizing factors as, for example, the importance of social leadership or the effect of daylight period or climatic factors (37). Cooper et al. (38) hypothesized cow behavior to be more synchronized at pasture due to an increased risk of predatory attack. Another self-evident explanation, especially regarding fattening bulls, could be the housing environment: They are generally fed using an *ad libitum* feeding regimen with an animal/feeding-place ratio of up to 2:1 (13). Therefore, the manger space generally does not permit simultaneous feeding of all animals per pen. In the present study, the manger space of 4.85 m per pen should provide at least six feeding places based on the recommendations of 75 cm feeding space for fattening cattle with a weight of more than 650 kg (13). However, the most frequently observed feeding condition showed only one to three bulls feeding simultaneously. The averaged percentage of animals feeding per pen never exceeded 20% of the bulls. This feeding behavior with animals feeding mostly alone or in groups of two to three is consistent with the results of other authors concerning fattening bulls fed *ad libitum* (21, 39). Gottardo et al. (39) compared two different manger spaces of 60 cm and 80 cm and observed that the bulls mostly visited the manger alone or in pairs, independent from manger space. Similar observations of a widely spread out feeding behavior with a low number of feeding animals at any time of the day exist for non-lactating dairy cows fed *ad libitum* (40, 41). In contrast, Longenbach et al. (42) found a strong motivation to visit the manger at the same time in heifers being subjected to restricted feeding. Based on these studies, it seemed likely that the motivation of cattle to synchronize their feeding behavior is reduced by the permanent availability of feed allowed by the *ad libitum* feeding regimen. In the present study, a limitation of manger space seems unlikely to be the reason for bulls mostly feeding alone or in small groups. In addition to the available manger space, this is confirmed by the fact that no animals seemed to be suffering from restricted access to feed. This is indicated by the satisfactory growth performance and BCS results as well as the varying order of bulls feeding after feed delivery.

As the present study did not imply a control group of bulls with a lower feeding frequency, the effects of feeding frequency on feeding behavior and growth performance could not be affirmed or definitely ruled out. However, growth performance was satisfactory and the observed percentage of time spent feeding comparable to other studies. The bulls spent an average of 9.9%, i.e., 107 min feeding per 18.5 h-period. Cozzi and Gottardo (21) observed feeding behavior in bulls during an average of 89 min per day and in a study of the Bavarian State Research Center for Agriculture, bulls spent an average of 95 min per day at the manger (43). Only Haskell et al. (44) observed higher daily feeding durations of an average of 133.4 min in fattening bulls. To our knowledge, further studies on fattening cattle do not exist and the higher feeding durations described for dairy cattle are presumably not transferable to fattening cattle due to highly divergent nutrient requirements. Further studies with experimental designs including control groups are required to investigate the effect of feeding frequency on feeding time in fattening cattle. However, an increasing effect of a higher feeding frequency in fattening cattle may be possible. In dairy cattle, such an effect has already been described by several authors (2, 8, 45).

Negative effects of an increased feeding frequency were reported by Phillips and Rind (46): They compared productivity and behavior of dairy cows fed once or four times per day and concluded that frequent feeding disturbed the cows and reduced milk. Similarly, Mäntysaari et al. (47) found an increased restlessness and decreased lying in dairy cows fed five times per day in comparison to cows fed only once per day. In the present study, there was a clear period of increased lying and decreased feeding activity during the night and early morning hours. This is consistent with cattle being diurnal feeders with a nocturnal resting period (6, 7). Furthermore, simultaneous lying of all bulls per pen occurred in all groups although the feeding behavior showed no clear synchronization patterns. More than 80% of the bulls per group were observed lying simultaneously during more than 20% of the total observation time, while during more than 30% of the total observation time 61–80% of the bulls were lying simultaneously. Consequently, despite the low degree of synchronization in feeding behavior, the lying behavior was highly synchronized in conformance with the natural behavior of gregarious animals such as cattle (36, 48). The high degree of synchronization is in accordance with studies on synchronized lying in dairy cattle with percentages of more than 80% of cows lying simultaneously occurring never or only during short periods of time (40, 41, 49). Individual measurements of lying behavior can also be interpreted as consistent with literature values. The mean lying bout duration of 63 min lies within the range of observed mean lying bout lengths in fattening bulls described by Reiter et al. (50) and Absmanner et al. (51). Literature values for the lying duration of bulls fluctuate around 800 min per day divided into 9 to 18 lying bouts (50–52). In the present study, bulls spent an average of 62.7% or 11:17 h (677 min) of the 18.5 h-period lying divided into 11 bouts. However, the highest percentages of lying animals were observed during the night and early morning hours that were excluded from the individual analysis. Therefore, the total lying duration of the bulls in the present study as well as their mean number of lying bouts can be expected to be considerably higher per 24 h-period than in the studied 18.5 h-period. Thus, the high feeding frequency in the present study did not seem to disturb the animals' lying behavior.

In the housing of fattening cattle, the delivery of fresh feed is usually performed only once to twice per day. Feed push-ups between feed deliveries ensure continuous access to feed, but lack the stimulating effect on feeding activity provoked by the delivery of fresh feed (22, 53). Furthermore, cattle are selective feeders (6). Cozzi et al. (34) observed diet selection towards more structured particles in bulls occurring 8–16 h after feed delivery. Such feed selection could have detrimental effects on nutrition, as feed quality possibly gradually declines with increasing time after feed delivery. Especially low-ranking animals without access to feed directly after feed delivery would possibly suffer as a result of always receiving pre-selected feed of reduced quality. To compensate this process and ensure not only constant feed availability, but also constant feed quality at any time of the day, a higher number of feed deliveries widely spread out across the daylight hours seems to be an efficient method. An observation of the present study possibly confirming this hypothesis is the absence of clear peaks in feeding activity after feed delivery. Apparently, there was no need for the animals to feed directly after feeding, this possibly indicating a constant feed availability and quality at any time. Studies on cattle fed only once or twice per day in general report such peaks in feeding activity directly after feed delivery (21, 22, 45, 54). Similarly, Mäntysaari et al. (47) observed peaks in feeding activity after feed delivery in dairy cows fed once per day, whereas the control group of cows fed five times per day tended to feed quite evenly after each delivery. Another interesting observation of the current study is the increased feeding behavior of the bulls during the afternoon and evening, reflecting the natural feeding pattern of cattle to take their most intensive meal of the day in the evening (6). The aim of accumulating a sufficient amount of feed to digest during the night seemed to be achieved as the feeding activity during and after the first feed delivery of the day was quite low. Therefore, the bulls were not starving after the night hours without delivery of fresh feed. These observations confirm that feed availability and quality in the evening were satisfactory. Furthermore, several feed deliveries performed in the evening as in the present study obviously promote the natural behavior of cattle to feed more in the evening. In contrast, the practice of feeding bulls only once per day in the morning may not be adequate to satisfy the nutritional and behavioral needs of cattle, given this natural feeding pattern. As feed quality is possibly reduced in the evening, bulls may not be able to accumulate a sufficient amount of nutrients for the night. This could lead to a reduction in growth performance as well as limitations of animal welfare.

The acquisition of an AFS is associated with a high initial capital investment. According to Haidn and Leicher (55), the investment calculations for an AFS in dairy cattle farms with 80 to 120 cows range from EUR 172,000 to EUR 250,000. To our knowledge, no studies on the economic efficiency of using AFS in fattening cattle exist. However, in dairy cattle, adding an AFS was shown to be justified economically despite the high initial investment due to a significant reduction in working time (1, 56). Bisaglia et al. (56) calculated a reduction of 100 min working time per day in comparison to conventional feed-mixer wagons for a herd of 120 dairy cows. Further research is required to assess the economic efficiency of using AFS in fattening cattle. However, as fattening farms generally house high numbers of animals, time-saving effects are possibly comparable to those observed in dairy cow systems.

The aim of the present study was to generally describe the behavior of fattening bulls fed six times per day with an AFS. The wide distribution of the feeding activity over the course of the day, the bulls mostly feeding alone or in groups of two or three as well as the increased feeding activity in the afternoon and evening emphasize the importance of constant feed availability and quality at any time of the day. All measures analyzed within the present study confirmed the ability of a high number of six feed deliveries per day to offer such a consistency. Indicators for negative effects of the high feeding frequency such as increased restlessness or restricted lying behavior were not observed. Therefore, the observations indicate that a high number of feed deliveries per day may not impair animal welfare. Clear conclusions regarding positive or negative effects of high feeding frequencies in comparison to conventional frequencies of one or two daily feed deliveries cannot be drawn from this study, as there was no control group. To draw such conclusions, further studies focusing on influences of feeding frequency on behavior and performance of fattening bulls are required.

## Conclusion

A higher number of feed deliveries distributed over the daylight hours, as it is possible using AFS, seems to be an effective method for ensuring constant feed availability and quality at any time of the day. The importance of this consistency is stressed by the tendency of cattle to spread out their feeding activity over the whole course of the day as well as the observation that they fed mostly alone or in groups of two to three.

## Data Availability Statement

The datasets generated for this study are available on request to the corresponding author.

## Ethics Statement

Ethical review and approval was not required for the animal study because it did not involve a prospective evaluation or laboratory animals and only non-invasive procedures were applied. It was reviewed and it received approval from the Animal Welfare Officer of the University of Veterinary Medicine Hannover, Foundation (reference: TVO-2017-B5). The study was carried out in accordance with the German legislations, the German Animal Welfare Act [German designation: TierSchG (11)], national requirements for animal husbandry [German designation: TierSchNutztV (12)], the Animal Protection guideline for Fattening Cattle of Lower Saxony (13) as well as the Council of Europe Convention on the protection of animals kept for farming purposes and its recommendations concerning cattle (14).

## Author Contributions

LS and BS contributed to conception and design of the study. LS organized the database and wrote the first draft of the manuscript. LS, NV, NK, and BS performed the statistical analysis. NV, NK, and BS reviewed and edited the manuscript. All authors contributed to manuscript revision, read, and approved the submitted version.

### Conflict of Interest

The authors declare that the research was conducted in the absence of any commercial or financial relationships that could be construed as a potential conflict of interest.
